# New York State Veterinary Medical Society

**Published:** 1892-09

**Authors:** N. P. Hinkley

**Affiliations:** Sec’y.


### SOCIETY PROCEEDINGS.

*New York State Veterinaay Medical Society.*—The New York State Veterinary Medical Society held its semi-annual meeting at the Vanderbilt House, Syracuse, N. Y., Thursday, July 14th, 1892.

Owing to the unavoidable absence of the President, Dr. R. S. Huidekoper, Dr. John Wende, Vice-President, occupied the chair, and called the meeting to order at 10 A. M.

The roll-call, by the Secretary, showed the following members present: Drs. John A. Bell, W. L. Baker, Charles Cowie, J. M. Chase, O. B. French, George Cowland, Wilson Huff, N. P. Hinkley, E. D. Hayden, E. B. Ingalls, C. D. Morris, Wm. Machan, L. A. Robinson, D. K. Seltzer, John Wende, L. E. Willyoung, J. A. Geming, A. H. Ide, R. A. McLean, A. Drinkwater. The following members were absent: Drs. R. S. Huidekoper, George H. Burns, R. R. Bell, James Carnite, W. H. Carpenter, W. A. Conklin, C. B. Comstock, W. G. Hollingworth, Benj. Howes, A. L. Hunter, J. G. Hill, M. J. Henderson, G. P. Jeffrey, H. C. Klicker, Wm. Kirk, W. G. Dodds, D. Leary, Prof. A. Liautard, Prof James Law, Drs. W. E. Langford, A. N. McQueen, G. H. Moulter, H. McWhmine, L. McLean, M. M. Poucher, H. E. Rowell, F. A. Rich, G. H. Roberts, Wm. Somerville, Jr., R. M. Somerville, H. Sutterby, J. K. Sutterby, F. Sutterby, W. S. Stevenson, G. H. Summerfeldt, P. K. Sidebottom, Wm. Somerville, Jas. Whytock, F. E. Williams, J. D Whytle, E. A. Wieland, H. S. Wende, R. S. Austin, H. W. Skerrit, F. Morrow, W. J. Wadsworth, V. L. James. Some of the above came in later in the day.

Dr. R. A. McLean informed the Society that President Huidekoper had been ordered to Homestead, Penn., being Lieut. Col. and Surgeon in Chief of the Division National Guard of Pennsylvania.

Dr. John Wende then read his address on the "Treatment of Scabies."

Dr. Hinkley—I think Dr. Wende's paper a very good one, and ought to bring forth an interesting discussion. Dr. Morris—I move that the paper be laid over for discussion until later in the meeting. Seconded by Dr. Mahaney; carried.

The reading of the minutes of the last meeting were dispensed with as they were published in both of the veterinary Journals of the State.

Dr. McLean—I move that the minutes of the last meeting as published in the *American Veterinary Review* and the JOURNAL OF COMPARATIVE MEDICINE be accepted and passed on. Motion seconded and carried.

Dr. Wende—The Board of Censors will now receive applications for membership. The following applications were received and acted upon by the Board of Censors:

W. E. Stocking, V. S., Eagle Harbor, N. Y.; Edward J. McLeod, V. S., Buffalo, N. Y.; James A. Pendergast, V. S., Phœnix, N. Y.; Lewis I. Palmer, V. S., Scottsville, N. Y.; Geo. J. Cangloff, D. V. S., Buffalo, N. Y.; E. H. Nodyne, V. S., Sodus Point, N. Y.; James McKee, V. S., Stapleton, S. I.; Saml. Somerville, Jr., V. S., Buffalo, N. Y.; Wilson S. Corlis, V. S., Carthage, N. Y.

Dr. Wende called the meeting to order, and Dr. McLean introduced Dr. Roberts, a medical man, who was accorded the floor and delivered a very interesting address, reciting some of his experience with tuberculosis during the past few years, saying that he received an inquiry some two years ago regarding Koch's treatment, and he wrote a criticism, which appeared in the *Chicago Inter-Ocean* at the time. He said that 90 per cent. of the M. D.'s claim that tuberculosis is produced by a germ, while he knows it to be a fact that it is communicable between animal and man. Speaking of the quarantine on cattle in Massachusetts, going to Maine and New Hampshire, he said that every animal must have a veterinary surgeon's certificate before allowed to leave the State of Massachusetts. He also said that in Syracuse there were two hundred deaths from consumption in the year of 1891. He thinks consumption is caused by tuberculosis from animals, and claims that people who are susceptible to consumption will take it from the dust of the spittle of the animal. In the New England States more people have consumption than in other States, because they are taken from school at the age of fifteen years and placed in factories to work, where they are compelled to breath the dust which causes this fatal disease.

By referring to the statistics of the different States you will find that the death rate from this disease decreases as we go west and south, while in some parts of New Mexico people have been cured after residing in the climate for a time. The air down there being pure enough to cure meat when hung out doors

Dr. Hinkley—Do they have tuberculosis in cattle in New Mexico?

Dr. Roberts—No. Dr. McLean—I found that steers coming from the west or south were comparatively free from tuberculosis, while in the New England States at least 33 1-3 per cent. were affected. During 1886 and 1887 there were three times more of deaths from tuberculosis than any other disease in the City of New York. I know of one family who were said to be suffering from consumption caused by using the milk from a pet Jersey cow that had tuberculosis. I think people who are susceptible to consumption are liable to have it by using milk from a diseased cow. A bill was brought before the Legislature regarding this matter, but when brought up for action was not acted upon. I do not believe there is any pleuro-pneumonia from Virginia to New York now since it was stamped out a few years ago.

Dr. McLean then cited his experience in this matter.

Dr. Roberts—According to the report of the city Board of Health of Boston the number of deaths from consumption is less, while those suffering from bronchial troubles are increasing. I think the government ought to take the matter in hand. Dr. Morris—I find that out of ten head brought in to be slaughtered five or six will be found suffering from tuberculosis, and that they are mostly from the east. Dr. John Wende—I make a motion that the members of this society tender their thanks to Dr. Roberts for his very able and interesting address. Seconded and carried. Dr. Roberts—I find in my travels through the New England States that 75 per cent. of the population are suffering from consumption. These people would be glad to leave this part of the country for the west and south, but as they are a working class without money they are unable to do so. I represent an Aid Society established for the purpose of assisting these people and would be pleased to receive any offerings that might be made.

Dr. McLean—I think that in the office where Dr. Morris is located they are able to stop the sale of cattle affected with pleuro-pneumonia, but cannot stop the sale of those affected with tuberculosis. Dr. Morris—This is so; but I think the government will take the matter in hand. Dr. Robinson—I found a case where the animal had tuberculosis, and the affected parts weighed twenty pounds or more. I gave it to the Health Commissioner of Buffalo, who has it preserved. I am told that this class of meat is cooked and eaten by the Polacks of Buffalo. Dr. McLean—I do not think there is as much danger in diseased meat as there is in the milk from a diseased cow. Dr Hinkley—We are discussing diseased meat which is always cooked before use, thereby avoiding the danger to a certain extent, while milk is generally used in its natural state I think the milk ought to be boiled at all times to destroy the bacilli as far as possible. Dr. Morris—The medical men recommend the use of condensed milk to avoid the dangers of bacilli.

Dr. Wende—The Secretary will please read the names of the applicants for membership who have been accepted by the Board of Censors. All whose names have been given were accepted.

Dr. Robinson.—I move we make the gentlemen members of our Society. Seconded ; carried.

Dr. Wende—Any unfinished business ? Dr. Morris—No

Dr. Wende—We will listen to report of the Committee on Arrangements. Dr. Hinkley—As a member of that Committee, would say we made the best arrangements we could. Dr. McLean—I move the report be accepted and Committee be tendered a vote of thanks Carried

Dr. Wende—Committee on Publication please report.

Dr. Hinkley—I will include the report in my annual report, and hope the Secretary will be excused from acting on all the committees in the future.

Dr. Wende—Committee on Legislation please report. Dr. Morris—The Committee have reported from time to time. I hoped to be able to furnish some new reports regarding this matter, but am unable to do so. It was a hard task to get the bill before the members of the Legislature. The more the question was brought before them the more they were found to be in favor of it. I found the most opposition was from Dr. Goldberg, member of Assembly, who asked if we could find among the profession of this State men who were able to dictate to the balance of the profession throughout the State. But the main objection was want of money. Mr. Duffy wanted $500 to pass the bill through, and we failed because we did not have the money to give them. I think if we cannot get it in this State we can get it from the United States, as all Western States have better laws in this matter than we have in New York State. I think we can do more in a higher authority and get more than in the Legislature of New York State. No matter how small an amount we asked for, it would be refused on general principles, in not passing it before. We have schools that are qualifying men in a course of one year; these schools ought to be done away with.

Dr. Morris offered the following resolution :

It being the object and desire of this society to lend its influence and individual energy to promote the best welfare, to build up and sustain the highest standard possible in our honorable profession, to watch with jealous care the approach of malicious influence emanating from societies or institutions which are holding out inordinate inducements to men of limited education, claiming to thoroughly equip and place them before the public in an acceptable manner upon short time as qualified veterinarians, the tendency of which only drags down and degrades the character of the profession, and that we believe the standard should be made higher and more uniform in character, and which we believe would be effectual only through centralized authority.

*Be it resolved:* That this society, with one accord, ask of the United States Veterinary Medical Society, at its next annual meeting, that steps be taken by the committee on legislation to seek by legislative enactment through Congress, the establishment of a National Board of Examiners with authority to confer the diploma of "The Veterinary Regents of America," and that the degree so conferred shall be final, and the only recognized certificate of proficiency to practice the art in the United States.

Dr. Morris spoke very favorably of having it offered at the meeting of the United States Veterinary Medical Association, to be held in September, 1892, and acted upon, as great benefits would be derived by having a United States Board of Regents established which would compel all veterinarians to pass the examination of this board, thereby making a universal standard throughout the United States. Dr. Wende—Have any of the members any remarks to make on the resolution offered by Dr. Morris? Dr. McLean—It would not be constitutional to pass such a law as Dr. Morris suggests. It is a stepping stone to establish a National School for Veterinary Medicine, and we cannot do it. I move the report be received. Motion seconded and carried.

Dr. Morris—I do not agree with the doctor, judging from our experience when we started. Prof's Law and Liautard say it is constitutional. I prefer it should be worked for New York State alone and would like to see it succeed, but it will take money to do it. Dr. McLean—I think the bill as presented was all right, if we could get it. But there cannot be general laws that will interfere with state laws. Dr. Morris—I think we can get it if every man will work for his Congressman. I agree with Dr. McLean, let every man see his Senator and Assemblyman I had to face one hundred letters from empirics when at Albany, and I cannot express a very favorable opinion of Dr. Goldberg as a professional man.

Dr. Baker—The member from my district supported the bill.

Dr. McLean—Kings and Queens counties will support the bill.

Dr. Hinkley—I am fully in accord with the bill, but think we ask too much. Let us ask for a distinction to be made between the veterinary surgeon and the farrier, and get it passed. I would like to see the members of this society attend the meeting more than they do and not stand back and ask for protection, but come forward and work on the committees. Dr. McLean—It should be made a cause for punishment if they do not do so. Dr. Morris—Some members of the Legislature think we ought to protect the empiric and help to raise him instead of asking for laws to compel him to come up to the standard. It was Dr. Goldberg who helped to a great extent to defeat the bill. Dr. McLean—I think we ought to amend the present law by giving the State Society power to prosecute all empirics who come here to practice. Dr. Hinkley—Do you want an amendment to the present law that will compel all veterinary surgeons to register? Dr. McLean—Yes. And give the State Society power to prosecute all who are not registered. Dr. Hinkley—I do not think we can punish them, as no one will do it for us. Dr. Baker—The fine for not registering is $200, as the law stands now.

Dr. Jno. Bell—I make motion to adjourn until 2 P. M. Carried.

AFTERNOON SESSION.

Dr. Jno. Wende—Is there anything further concerning the matter of legislation? Dr. Jno. A. Bell—I move the report of the committee be received and approved. Dr. O. B. French—Second the motion. Carried. Dr. McLean—I move that the committee be continued and that they prepare an amendment to the present bill, giving power to the State Society to prosecute all who are practicing without complying with the law of 1886, and prosecute all who swear falsely for perjury, fine them, one-half of fine to go to the society. Motion seconded and carried.

Dr. Morris—Will the secretary read the names of the committee on legislation?

Dr. Hinkley - The following are the members of this committee: Drs. R. A. McLean, C. D. Morris, R. R. Bell, N. P. Hinkley, Geo. H. Berns, and Prof's Jas. Law, A. Liautard.

Dr. Wende—Committee on by-laws and constitution report.

Dr. McLean—We have not been able to make any progress as yet. I, as chairman, have notified the members of the committee to meet once at Albany and once at Syracuse, but being unable to get them together, we could not act. I move the report be accepted and the committee be discharged. Dr. Morris—I amend that we accept the report with thanks to the committee Seconded. Carried. Dr. Hinkley—I make a motion the vice-president be empowered to appoint a committee of five on revision of by-laws and constitution. Seconded and carried.

Dr. Wende—I appoint the following as a committee on revision of by-laws and constitution: Dr. R. A. McLean, Chairman, Brooklyn; Dr. L. E. Willyoung, Albion; Dr. Jno. A. Bell, Watertown; Dr. C. D. Morris, Brooklyn; Dr. W. L. Baker, Cortland.

Dr. McLean—I would suggest that all essayists write their papers from their own experience and not copy from books or journals.

Dr. Wende—Any new business? Dr. Hinkley—I have a letter from Dr. W. Horace Hoskins, Secretary of the United States Veterinary Medical Association. He is very anxious that this society should be well represented at their next meeting to be held at Boston, Mass., in September, 1892, and I would advise all who are not members of that society to send in their applications at once. It will be a three days' session and I am sure you will all enjoy it very much. The important question of how we shall visit the International meeting in Chicago in 1893, will be freely discussed and decided upon. Also the question of the standard of members of the profession who will be allowed to become members of the association. It will be a great benefit to all of you to attend as most of the members of this association are very talented men

Dr. Morris—I want the society to make as good a showing as possible at this meeting, as important questions will be discussed.

Dr. Willyoung—Elect as many delegates as will attend.

Dr. Wende—Will the gentlemen who expect to attend, stand up. The following were elected delegates to attend the meeting of the United States Veterinary Medical Association, to be held at Boston, Mass., in September, 1892: Drs. E. J. McLeod, N. P. Hinkley, C. D. Morris, Jno. A. Bell, Jno. Wende, W. L. Baker, J. M. Chase, R. A. McLean.

Dr. Morris—I coincide with Dr. McLean's idea of writing papers on facts as they occur in our practice. I would like good advice on castration in lower animals. I try every manner possible to make it a successful operation by keeping everything clean and using antiseptics in every instance and yet the colts will die, while on the other hand the empirics will go out and castrate a colt on a manure heap, using dirty ropes, dirty cords, and everything about him smeared in dirt, and his case will come out all right. If any of you can tell me the reason why, I would be pleased to learn. Dr. Wende—I have had a large experience in castration ; operated on 300 last year and lost three with peritonitis, at the same place I lost two this year. In one instance last year, I operated on four for one man; one was sent to the pasture and recovered nicely, three were kept near the barn and two of them died, but I saved the third one. Dr. Cowie—I think quacks lose more than we know of, while if we lose one our reputation is at stake and it is reported.

Dr. Chase—I want to ask Dr. Wende what he thinks caused the death of his colts? Dr. Wende—In one case where a quack operated the year before, I think the colts were trifled with. I think where other diseases are being treated at same place, their death will be caused by germs. and that the germs can be carried in the clothes from place to place. Dr. McLean—I will not castrate any more in my hospital.

Dr. Hinkley—I will not castrate any more in my hospital, and had decided upon this some time ago. Dr. Morris cites a case where he believes he carried tetanus from a patient to a colt recently operated on and it died. Dr. Chase—Is there any one present who has castrated without losing any? Dr. W. Huff—I have never lost a case. Dr. W. L. Baker—I can say the same.

Dr. Chase—I make motion that Dr. Wende appoint a committee of two to choose subjects for the members to write on and discuss at the next meeting. Motion seconded. Carried.

Dr. Wende—I appoint Dr. R. A. McLean and Dr. J. M. Chase. The subjects chosen were: Influenza and its complications; antiseptics, their uses and modes of application; tuberculosis, especially its generation.

Dr. Hunter—I would like to hear the opinion of the members on purpura-hæmoragica. I was called to a case where a good mare had a swelling as large as the fist about half way down the Trachea. She looked well, appetite was good. At first I thought it was a bruise caused by the manger, after a week I was called again and found the neck badly swollen all the way to the thorax. She could drink water well, but the food came out of the nostrils when trying to swallow. I gave aconite, and fomented the swellings, after a few days she appeared all right. The symptoms of purpura were prevalent. I gave ergot, chlorate potash and tincture iron. The head was swollen, but after a few days symptoms all disappeared and she seemed all right; on following Sunday I was called and found her in pains similar to colic, gave a hypodermic injection of morphia which quieted her, but she died that night. I held post mortem the next afternoon. Did not find any injuries internally, but on taking off the front leg I found an abscess on the inside of the scapula about the size of a two-quart pail. I had three cases, all presented different symptoms. There were abscesses in two of them but not any in the third. Have had several cases during past two months. Have the best results from treating with nux vomica. Dr. Morris—I have good success by evacuating the bowels and treating locally with stimulants and tonics. Dr. Robinson—I had two cases which I treated successfully with powdered and fluid extract nux vomica. Dr. Hinkley—Have any of you derived any benefit from giving chloride potash in purpura? Dr. Willyoung—Only in quantities not sufficient to produce a laxative condition of the bowels. Dr. Hinkley—Would you advise using it to produce a laxative effect in purpura? Dr. Willyoung—No, sir. Dr. Morris—I like iodide potash best. Dr. Hinkley—I have treated twenty-five to thirty cases without loss of any; used powdered nux vomica, quinine and tincture iron, and used turpentine for the kidneys, if needed. I use ice instead of fomentations. Dr. Baker—I use iron and quinine successfully. Dr. Morris—I think Dr. Hunter treats scientifically. I have used ice water with good results. Dr. Hunter—How do you use ice? Dr. Hinkley – Use a packing of ice and bran or salt and bind on. I think Dr. Hunter's cases would have proven fatal under any treatment. I know of vetrinarians in Cleveland who use per oxide hydrogen successfully. I have had no success with it in purpura. Dr. Drinkwater—Have any of you had a case of purpura in dogs? I have a case; the symptoms are similar to those in a horse. Dr. Hinkley—Has the dog been sick previously to this? Dr. Drinkwater—Yes; two or three weeks with distemper. I have not had time to study up the case thoroughly before coming to this meeting.

Dr. Baker—I have several cases of a disease in the regions of the horns of cattle caused by a small black fly. Do not find a remedy as yet. Dr. French—Have had several cases and used tar with good results. I do not consider it a serious trouble. Dr. Palmer—have treated several cases successfully with tar.

Dr. McLean—I make a motion that all members who are two years in arrears must pay up before the next meeting or have their names dropped from the roll, and that the Secretary notify them to this effect. Seconded and carried.

Dr. McLean—I make a motion that a special assessment of $2.50 be made on each member of this society to liquidate the indebtedness of the society. Dr. French—I second the motion. Carried unanimously.

Dr. Robinson—I make a motion that the matter pertaining to Drs. Wm. Somerville, Wm. Somerville, Jr., and Robt. M. Somerville be brought before the Board of Censors to be acted upon. Dr. Willyoung—Second the motion. Carried. Dr. Wende—Will the Chairman of the Board of Censors report what action has been taken in this matter. Dr. John A. Bell—We have not received any correspondence from the Drs. Somerville on this matter, consequently taken no action thus far. Dr. Wende—The Secretary will please read the correspondence on this matter. The Secretary read all the correspondence on the matter.

Dr. Wende—What action will the Board of Censors take?

Dr. S. Somerville, Jr.—I make a motion that the matter pertaining to Dr. R. M. Somerville be laid over until the next meeting. Dr. McLean—We ought to settle it now and not allow these matters to drag along. Dr. Wende—I appoint Dr. McLean to fill the vacancy on the Board of Censors during this meeting. Said vacancy caused by absence of Dr. James. Dr. Wende—The Board of Censors will retire to a private room in company with Dr. S. Somerville, Jr., to act upon the matter pertaining to the Drs. Somerville.

The following is the decision of the Board of Censors:

That the resignation of Wm. Somerville, Jr., as received be not accepted, that he be expelled from the New York State Veterinary Medical Society. That the cases of Wm. Somerville, Sr. and Robt. M. Somerville be held over until the next meeting and that no charges have been preferred against S. Somerville and son, and that Wm. Somerville, Sr., and Robt. M. Somerville be notified by the Secretary to appear before this Committee and confirm the explanation given to-day.

John A. Bell,
R. A. McLean,
J. M. Chase,
John Wende,
O. B. French,
*Board of Censors*.

During the special session of the Board of Censors quite a discussion took place regarding hernia in colts and calves and the different modes of treatment. Several members cited cases with which they had to deal with during their practice. The subject of the black fly, which has appeared among the dairy farms of this State, was brought up again for discussion, and several remedies were recommended, among them crude oil, fish oil, and carbolic acid, the latter recommended by Prof. Law.

Dr. Robinson gave a very interesting description of a case which he had in his hospital. A horse, seven years old, ran a stick into the body eighteen inches; during the treatment he made incisions in seventeen different places, and removed two quarts of pus from the sheath after six weeks treatment, using peroxide hydrogen, and salicylate soda, and quinine as a constitutional remedy; he made a good recovery.

A discussion regarding changing the place of meeting at different times, led to a vote which resulted in favor of changing, also the matter of having one meeting per year of two days' session, instead of two meetings of one day each. A vote resulted in favor of the former; this was done to get the opinion of the members present; the change cannot be made until after the revision of the by-laws.

Dr. McLean—I find that I can always learn somethin5 of value to me at these meetings. At the annual meeting the subject of Pyoktanine and its uses were discussed. Since then I have met with wonderful success in using it. I feel that I am indebted to the New York State Veterinary Medical Society for this. Dr. Bell—I have used it with equally good result. Dr. Wende—I have used it with good results where other remedies have failed.

Dr. Baker—How does Dr. Hinkley use pepsin in fistula-withers? Dr. Hinkley – I fill the cynus full of crystal pepsin, and cover the wound with absorbent cotton. I find that it eats its way through. I have found it very good in poll-evil also I have healed some with pyoktanine. Dr. Wende—I use spirits turpentine in fistula-withers and get good results. Dr. Chase—What becomes of the genus?

Dr. Wende—It fills up with granulations. I used it on my own horse six years ago and have had no trouble with it since.

Dr. McLean—Do you recommend the injection of one or two drams of turpentine into the sore? Dr. Wende—Yes. It will not hurt them. Dr. Gowland—I get good results from iodine one part, glycerine two parts. Dr. Bell—How long do you use turpentine? Dr. Wende—Until it is all healed. Dr. Chase—How much do you use? Dr. Wende—Enough to fill the sore. Dr. McLean—I was called in consultation in a case of fistula-withers, passed probe down 7½ inches, cut through and drew seton through. At the end of five days sent for me, I found a nasty discharge, took out seton, used injection of pyoktanine, made good recovery.

Dr. Bell—I would like to have a treatment for locomotor ataxia. Dr. McLean—Would suggest two ounces of lead and powder.

It was getting late, and as all business had been finished, it was moved and seconded that the society tender a vote of thanks to Mr. Fife for his kindness in granting the use of the Assembly room to the members of the society, and that we adjourn until the annual meeting, to be held in January, 1893, subject to a call by the secretary.

N. P. Hinkley, D.V.S., *Sec'y.*

